# Molnupiravir inhibits SARS-CoV-2 variants including Omicron in the hamster model

**DOI:** 10.1172/jci.insight.160108

**Published:** 2022-07-08

**Authors:** Kyle Rosenke, Atsushi Okumura, Matthew C. Lewis, Friederike Feldmann, Kimberly Meade-White, W. Forrest Bohler, Amanda Griffin, Rebecca Rosenke, Carl Shaia, Michael A. Jarvis, Heinz Feldmann

**Affiliations:** 1 Laboratory of Virology and; 2 Rocky Mountain Veterinary Branch, National Institute of Allergy and Infectious Diseases (NIAID), NIH, Hamilton, Montana, USA.; 3 School of Biomedical Sciences, University of Plymouth, Plymouth, United Kingdom.; 4 The Vaccine Group, Plymouth, United Kingdom.

**Keywords:** COVID-19, Virology, Drug therapy, Mouse models

## Abstract

The recent emergence of the SARS-CoV-2 Omicron variant of concern (VOC), which contains a heavily mutated spike protein capable of escaping preexisting immunity, identifies a continued need for interventional measures. Molnupiravir (MK-4482), an orally administered nucleoside analog, has demonstrated efficacy against earlier SARS-CoV-2 lineages and was recently approved for SARS-CoV-2 infections in high-risk adults. Here, we assessed the efficacy of MK-4482 against the earlier Alpha, Beta, and Delta VOCs and Omicron in the hamster COVID-19 model. Omicron replication and associated lung disease in vehicle-treated hamsters was reduced compared with replication and lung disease associated with earlier VOCs. MK-4482 treatment inhibited virus replication in the lungs of hamsters infected with Alpha, Beta, or Delta VOCs. Importantly, MK-4482 profoundly inhibited virus replication in the upper and lower respiratory tract of hamsters infected with the Omicron VOC. Consistent with its mutagenic mechanism, MK-4482 treatment had a more pronounced inhibitory effect on infectious titers compared with viral RNA genome load. Histopathologic analysis showed that MK-4482 treatment caused a concomitant reduction in the level of lung disease and viral antigen load in infected hamsters across all VOCs examined. Together, our data indicate the potential of MK-4482 as an effective antiviral against known SARS-CoV-2 VOCs, especially Omicron, and likely future SARS-CoV-2 variants.

## Introduction

Now in its third year, the coronavirus disease 2019 (COVID-19) pandemic has become characterized by the serial emergence of severe acute respiratory syndrome coronavirus 2 (SARS-CoV-2) variants of concern (VOCs) that rapidly and globally replace earlier, previously predominant strains. In November 2021, Omicron (B.1.1.529) emerged and rapidly replaced Delta (B.1.617.2), the predominant VOC at the time (1). Omicron has shown reduced pathogenicity (2) but enhanced transmissibility primarily associated with an increased ability to evade protective immunity associated with infection by earlier VOCs or through vaccination (3, 4). The emergence of VOCs with immune evasive ability poses a continuous and considerable threat to global control strategies, which are based on present spike-based vaccines as well as monoclonal antibody-based therapeutics for treatment of more severe COVID-19.

Molnupiravir (MK-4482) is an orally available antiviral nucleoside analog that targets SARS-CoV-2 polymerase fidelity rather than the spike protein (5). It has been authorized for emergency use against SARS-CoV-2 in high-risk adults (6, 7). Studies from early in the pandemic showed the ability of MK-4482 to suppress acute virus replication and associated disease in the Syrian hamster COVID-19 model (8) and transmission in ferrets (9). Both of these early studies were performed before VOC emergence became the norm and showed effect against the original Washington SARS-CoV-2 variant, which was still prevalent at this time. Later in the pandemic, efficacy in hamsters was shown against Alpha and Beta VOCs, which were emerging at the time (10). Results from recent in vitro studies suggest that MK-4482 retains activity against multiple VOCs including the more pathologic Delta and transmissible Omicron variants (11). However, experience with therapeutics targeting SARS-CoV-2 (12), as well as other emerging viruses (13), have shown a frequent disconnect between efficacy observed in vitro compared with therapeutic in vivo effect.

In the present study, we investigated the ability of MK-4482 to inhibit several SARS-CoV-2 VOCs in the Syrian hamster COVID-19 model. MK-4482 reduced viral RNA loads and significantly reduced infectious virus in the lungs for animals infected with the Omicron as well as Alpha, Beta, and Delta VOCs. Notably, MK-4482 also significantly reduced infectious virus in oral swabs and the upper and lower respiratory tracts in hamsters infected with a high dose of Omicron. Our study demonstrates the efficacy of MK-4482 against multiple divergent VOCs, including Omicron, and is likely an effective antiviral against future emerging SARS-CoV-2 variants.

## Results

**Figure 1:**
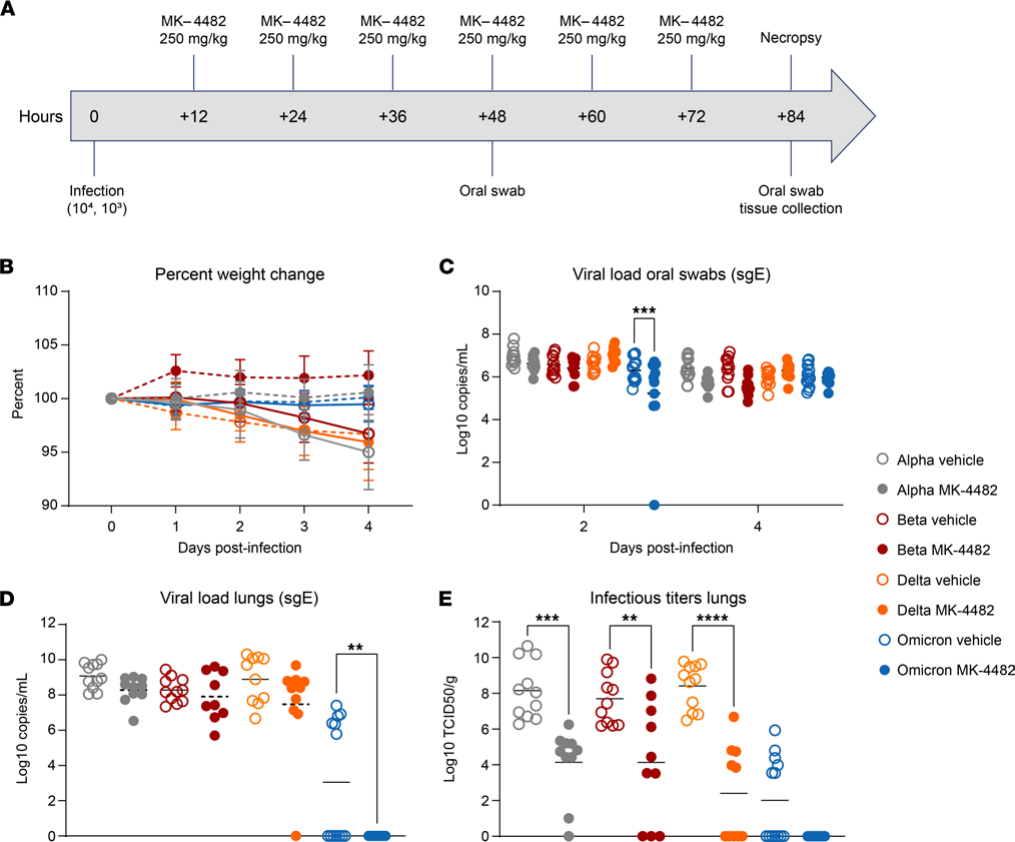
Efficacy of MK-4482 on upper and lower respiratory tract viral load and infectivity in hamsters infected with SARS-CoV-2 Alpha, Beta, Delta, or Omicron VOCs. (**A**) Experimental design. Hamsters (*n* = 11 vehicle, *n* = 10 treatment) were intranasally infected with 10^3^ TCID_50_ of the different SARS-CoV-2 VOCs. Treatment was started 12 hours after infection and continued every 12 hours. Oral swabs were collected at 2 and 4 dpi and animals were necropsied at 4 dpi for tissue collection. (**B**) Clinical presentation. Changes in body weight were recorded over the entire study period of 4 days. (**C**) Viral RNA load in oral swabs. Alpha, Beta, Delta, or Omicron RNA loads were determined by quantitative RT-PCR targeting sgE as a surrogate for replication and shedding. (**D**) Viral RNA loads in lung tissue. Alpha, Beta, Delta, or Omicron RNA loads were determined by quantitative RT-PCR targeting sgE as a surrogate for replication. (**E**) Infectious viral titers in lung tissue. Alpha, Beta, Delta, or Omicron infectivity was determined using a tissue culture infectious dose (TCID) assay and are presented as TCID_50_/g tissue. Statistical differences in viral load and infectious virus titers in each study arm were assessed by ordinary 1-way ANOVA (*P* < 0.05). *P < 0.05, **P < 0.01, ***P < 0.001, ****P < 0.0001.

Syrian hamsters were randomly divided into vehicle or MK-4482 treatment groups and infected intranasally with 10^3^ median tissue culture infectious dose (TCID_50_), as previously described (14), to test in vivo efficacy of MK-4482 against the SARS-CoV-2 Alpha, Beta, Delta, and Omicron VOCs. Treatment using 250 mg/kg MK-4482, administered by oral gavage, was initiated 12 hours after infection. Treatment was continued every 12 hours for the established total daily dose of 500 mg/kg (8). Hamsters were monitored daily for clinical signs, and oral swabs were collected 2 and 4 days post infection (dpi). At 4 dpi hamsters were euthanized and tissues were collected for viral RNA load and infectious titer determination (Figure 1A).

Hamsters in vehicle and MK-4482 treatment groups remained largely asymptomatic, with noticeable but not significant weight loss (3%–4%) in vehicle-treated animals over the 4-day study period (Figure 1B). Quantitative RT-PCR targeting subgenomic viral E gene RNA (sgE) in oral swabs and lung tissue was used to quantify replication in the upper and lower respiratory tract, respectively (15). sgE loads in oral swabs from vehicle-treated animals at 2 and 4 dpi ranged from 5 to 8 log_10_ copies/mL. The presence of detectable sgE loads in oral swabs of all infected hamsters, except for a single animal in the Omicron MK-4482 treatment group confirmed VOC infection with the exception of this single Omicron animal (Figure 1C). MK-4482 treatment resulted in lower replication in oral swabs for all VOCs except Delta, a result that was only statistically significant for Omicron at 2 dpi (Figure 1C). sgE loads in lung tissue ranged from 7 to 10 log_10_ copies/g for Alpha, Beta, and Delta in vehicle-treated groups, but they were notably lower (0–7 log_10_ copies/g) for Omicron (Figure 1D). MK-4482 treatment resulted in a reduction in sgE lung loads for all VOCs and was below the limit of detection for Omicron, a result that was statistically significant (Figure 1D). Similarly, infectious virus titers in lung tissue ranged from 6 to 11 log_10_ TCID_50_/g for Alpha, Beta, and Delta vehicle-treated groups and were significantly lower for Omicron (0–6 log_10_ TCID_50_/g) (Figure 1E). Infectious titers were substantially more reduced by MK-4482 than sgE loads. This was observed for all VOCs, with no infectious virus detected in lung tissue from any Omicron-infected hamster (Figure 1E).

**Figure 2:**
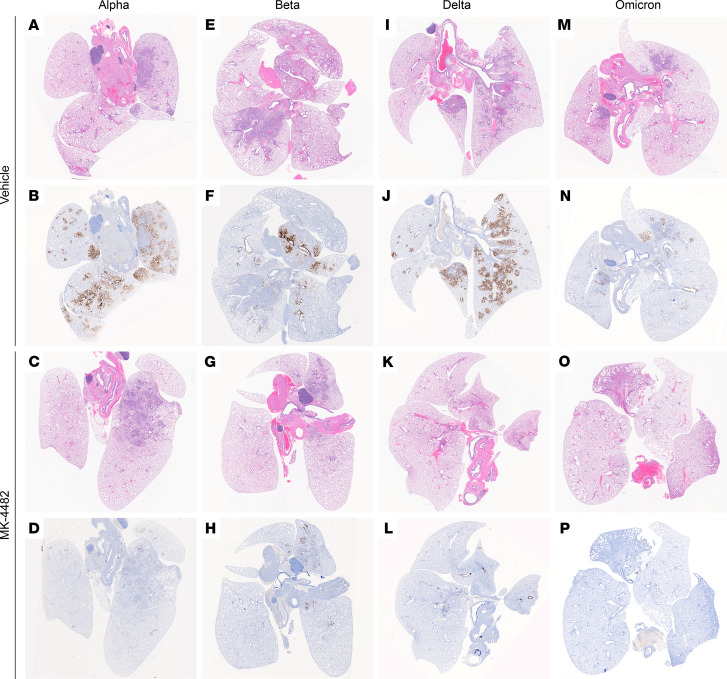
Efficacy of MK-4482 on lung tissue from hamsters infected with SARS-CoV-2 Alpha, Beta, Delta, or Omicron VOCs. Experimental design is shown in Figure 1A. Lung tissue was collected at 4 dpi and prepared for H&E staining and IHC using an antibody directed against the SARS-CoV-2 nucleocapsid protein. (**A**, **E**, **I**, and **M**) Lung histopathology. H&E staining of lung tissue from vehicle-treated hamsters infected with Alpha, Beta, Delta, or Omicron VOC, respectively. Areas of consolidation represent foci of bronchointerstitial pneumonia. (**B**, **F**, **J**, and **N**) Lung immunohistochemistry. SARS-CoV-2 nucleoprotein detection in lung sections from vehicle-treated hamsters infected with Alpha, Beta, Delta, or Omicron, respectively. Corresponding foci of immunoreactivity (brown color) are shown. (**C**, **G**, **K**, and **O**) Lung histopathology. H&E staining of lung tissue from MK-4482–treated hamsters infected with Alpha, Beta, Delta, or Omicron VOC, respectively. Areas of consolidation represent foci of bronchointerstitial pneumonia. (**D**, **H**, **L**, and **P**) Lung immunohistochemistry. SARS-CoV-2 nucleoprotein detection in lung sections from MK-4482–treated hamsters infected with Alpha, Beta, Delta, or Omicron, respectively. Corresponding foci of immunoreactivity (brown color) are shown. Whole lung sections are shown.

**Figure 3:**
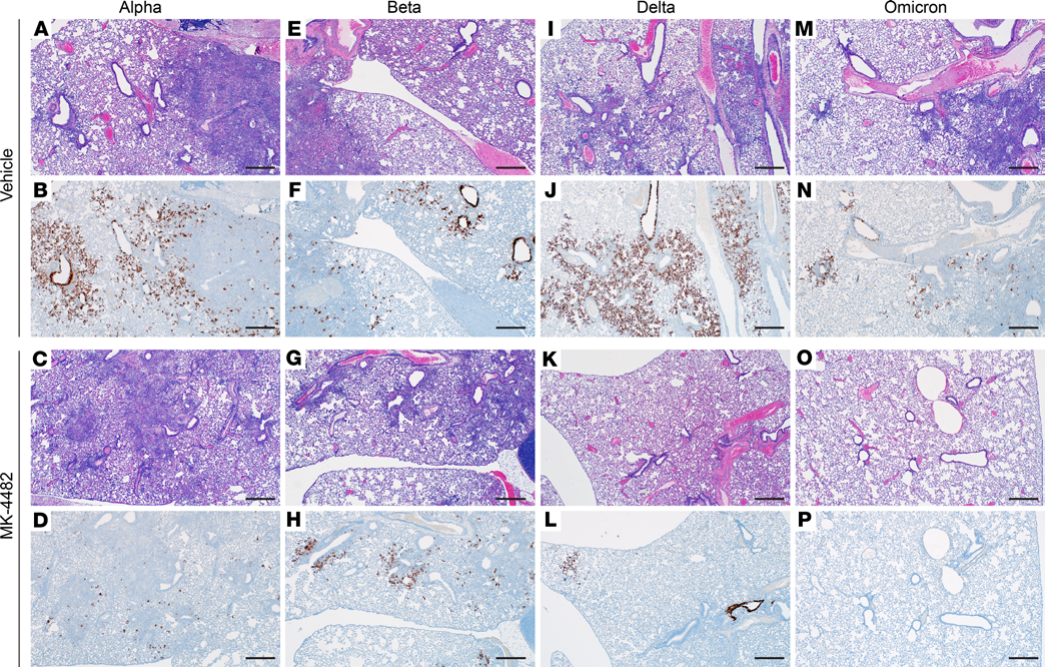
Efficacy of MK-4482 on lung pathology in hamsters infected with multiple SARS-CoV-2 VOCs. The experimental design is shown in Figure 1A. Lung tissue was collected at 4 dpi and prepared for H&E staining and IHC using an antibody directed against the SARS-CoV-2 nucleocapsid protein. (**A**, **E**, **I**, and **M**) Lung H&E staining (original magnification, ×40) of tissue from vehicle-treated hamsters infected with Alpha, Beta, Delta, and Omicron VOC, respectively, showing bronchointerstitial pneumonia and vasculitis. (**B**, **F**, **J**, and **N**) Lung IHC (original magnification, ×40). SARS-CoV-2 nucleoprotein detection in lung sections from vehicle-treated hamsters infected with Alpha, Beta, Delta, and Omicron, respectively, exhibiting immunoreactivity associated with areas of pneumonia (brown color). (**C**, **G**, **K**, and **O**) Lung H&E staining (original magnification, ×40) of tissue from MK-4482–treated hamsters infected with Alpha, Beta, Delta, and Omicron VOC, respectively, showing reduced bronchointerstitial pneumonia. (**D**, **H**, **L**, and **P**) Lung IHC (original magnification, ×40). SARS-CoV-2 nucleoprotein detection in lung sections from MK-4482–treated hamsters infected with Alpha, Beta, Delta, and Omicron VOC, respectively, exhibiting reduced or no immunoreactivity. Each panel shows a lung sections from a representative hamster. Scale bar: 500 μm.

Histopathologic analysis of lung tissues revealed minimal-to-moderate bronchointerstitial pneumonia for vehicle-treated hamsters infected with Alpha, Beta, and Delta VOCs (Figure 2). Omicron-infected animals showed only mild lesions, reflecting reduced virus replication of this VOC in the lower respiratory tract (16) (Figure 2). In general, lesions were as previously described (14). They consisted of bronchiolar and alveolar inflammation, most prominently around terminal bronchioles, with neutrophils, macrophages, necrotic debris, fibrin, and edema in associated alveolar spaces as well as variable amounts of septal inflammation and thickening (Figure 3). Other features included single and small clusters of necrotic respiratory epithelium in large and medium caliber bronchioles and vasculitis in locally associated blood vessels (Figure 3). Lesions were reduced in all MK-4482–treated hamsters, as is noticeable and best shown by the lower-magnification lung H&E overview staining (Figure 2) and also shown in the higher-magnification, detailed H&E-stained sections (Figure 3). Immunohistochemical labeling of affected and nonaffected bronchioles and alveoli was common throughout, but it was especially present at the margins of regions of pneumonia for vehicle-treated hamsters infected with the Alpha, Beta, and Delta VOCs (Figures 2 and 3). Labeling was only infrequently found with hamsters infected with the Omicron VOC (Figures 2 and 3). Immunoreactivity was consistently less frequent in MK-4482–treated hamsters for each group, with basically no immunoreactivity in the treated Omicron-infected hamsters (Figures 2 and 3).

**Figure 4:**
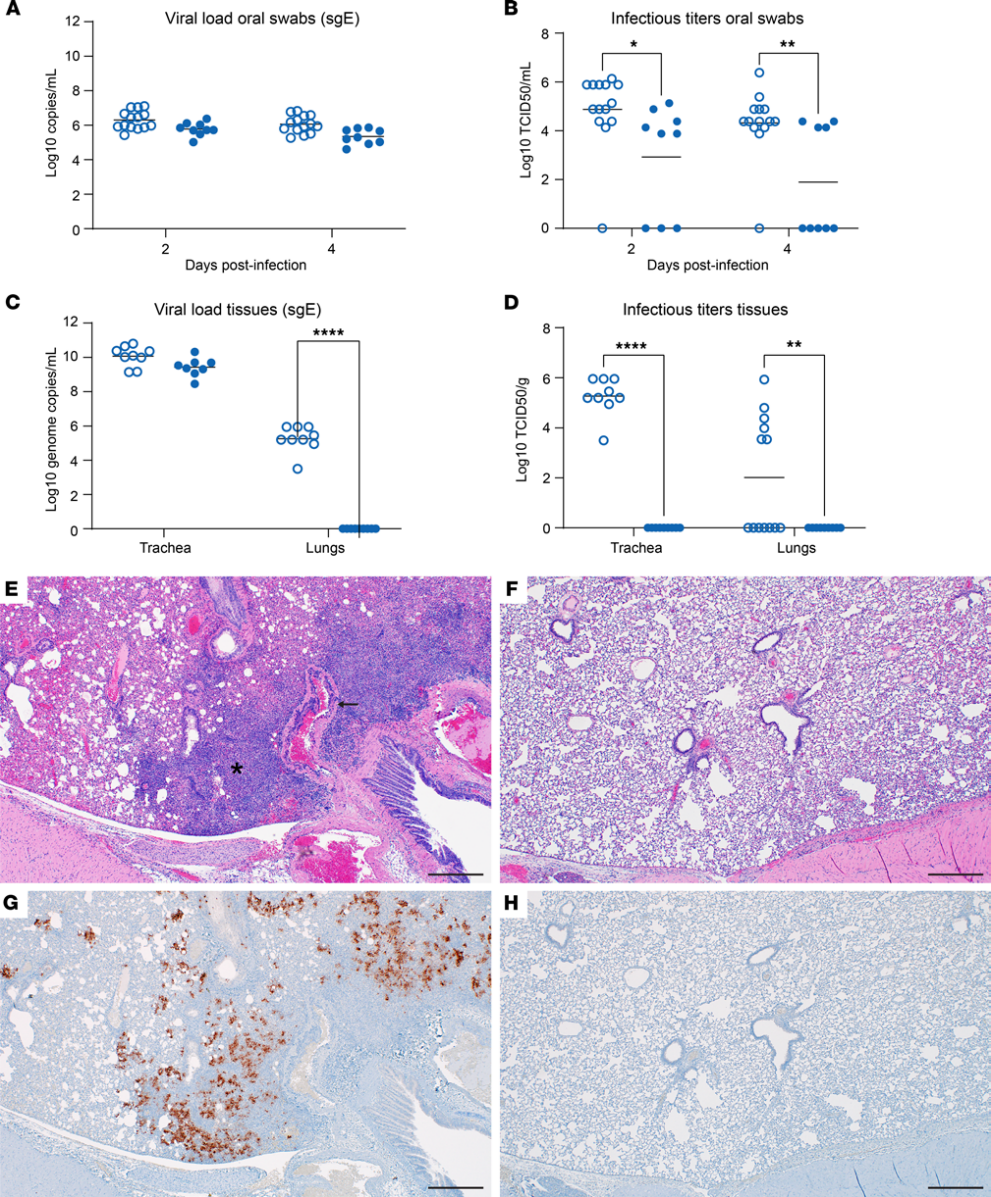
Efficacy of MK-4482 on upper and lower respiratory tract viral load, infectivity, and lung pathology in hamsters infected with high-dose Omicron SARS-CoV-2 VOC. The experimental design is shown in Figure 1A. For this experiment we used an Omicron challenge dose of 10^4^ TCID_50_ and collected tracheae as an additional tissue. (**A**) Omicron RNA load in oral swabs. Viral loads were determined by quantitative RT-PCR targeting sgE as a surrogate for replication and shedding. (**B**) Infectious Omicron titers in oral swabs. Viral infectivity was determined using a TCID_50_ assay and is presented as TCID_50_/g tissue. (**C**) Omicron loads in trachea and lung tissues. Viral loads were determined by quantitative RT-PCR targeting sgE as a surrogate for replication. (**D**) Infectious Omicron titers in trachea and lung tissues. Viral infectivity was determined using a TCID_50_ assay and is presented as TCID_50_/g tissue. Statistical analysis was performed in Prism 9. (**E**) Lung H&E staining (original magnification, ×40) of a vehicle-treated hamster infected with Omicron exhibiting a focus of bronchointerstitial pneumonia (asterisk) and vasculitis (arrow). (**F**) Lung H&E staining (original magnification, ×40) of a MK-4482–treated hamster infected with Omicron showing normal pathology. (**G**) Lung IHC (original magnification, ×40) of a vehicle-treated hamster infected with Omicron exhibiting frequent immunoreactivity associated with focus of pneumonia (brown color). (**H**) Lung IHC (original magnification, ×40) of a MK-4482–treated hamster infected with Omicron exhibiting absence of immunoreactivity. Each panel shows a lung section from a representative hamster. Scale bar: 500 μm. Statistical differences in viral load and infectious virus titers in each study arm were assessed by ordinary 1-way ANOVA (*P* < 0.05). *P < 0.05, **P < 0.01, ***P < 0.001, ****P < 0.0001.

We repeated the MK-4482 treatment study with a higher Omicron challenge dose (10^4^ TCID_50_) to account for the decreased replication of the Omicron VOC in lung tissue (16, 17). The study design remained the same as described above (Figure 1A), but trachea tissue was collected at 4 dpi as an additional target tissue. The higher Omicron challenge dose did not increase clinical disease severity and weight loss remained similar to that of animals challenged with the lower 10^3^ TCID_50_ dose (Figure 1B). At 2 and 4 dpi, sgE loads of oral swabs from vehicle-treated animals ranged from 5 to 7 log_10_ copies/mL; these loads were lower in MK-4482–treated animals (<1 log_10_ copies/mL), a difference that was not statistically significant (Figure 4A). Infectious virus in oral swabs from vehicle-treated animals ranged from 4 to 7 log_10_ TCID_50_/mL (Figure 4B); this was significantly reduced by several log_10_ following MK-4482 treatment (Figure 4B). sgE loads and infectious titers were also determined in trachea and lung tissues at 4 dpi. In contrast to that in the trachea, approximately half of the animals in the vehicle-treated group lacked detectable virus in the lung (by TCID_50_), consistent with lower replication of Omicron in this tissue in general (16). Remarkably, MK-4482 treatment reduced sgE loads and infectious virus to below the limit of detection in lung tissue of all animals, corresponding to an average reduction of more than 4 log_10_ (Figure 4C) and more than 2 log_10_ (Figure 4D), respectively. Interestingly, sgE loads were several log_10_ higher in trachea tissue (9–11 log_10_ copies/g) compared with lung tissue of vehicle-treated hamsters (Figure 4C). sgE loads were less than 1 log_10_ lower in trachea tissue from MK-4482–treated animals (Figure 4C). However, infectious virus in trachea tissue dropped precipitously from a median of 5 log_10_ TCID_50_/g in vehicle-treated animals to below the limit of detection in MK-4482 animals (Figure 4D). Histopathologic analysis of the lungs confirmed the findings on viral loads and infectious titers in lung tissue, showing reduced lesions (Figure 4, E and F) and no viral antigen (Figure 4, G and H) in the MK-4482–treated Omicron infected hamsters.

## Discussion

Since SARS-CoV-2 emerged in late 2019, there have been at least 13 variants of interest or VOCs circulating throughout the world (18). As each variant is likely to become more adapted at spread and avoiding preexisting immunity developed by previous infection or vaccination, refining existing and developing new treatment options becomes critically important for our response to the SARS-CoV-2 pandemic. In this study, we evaluated the efficacy of MK-4482 against several SARS-CoV-2 VOCs in the Syrian hamster model. MK-4482 was efficacious against all VOCs, with significant reduction of virus replication in the lower respiratory tract. This was particularly evident for the Omicron VOC, with absence of any infectious virus in trachea and lung samples. Thus, MK-4482 remains potent against SARS-CoV-2 variants, including Omicron, likely positively affecting outcomes for patients with COVID-19.

The effect of an antiviral intervention on virus shedding, a key driver of any epidemic and pandemic, is important. In contrast to SARS-CoV-2 replication in the lower respiratory tract, MK-4482 treatment only slightly decreased replication and shedding from the upper respiratory tract for the Alpha, Beta, and Delta VOCs. Interestingly, MK-4482 treatment significantly reduced replication and shedding of Omicron, indicating potent efficacy against the VOC driving the current SARS-CoV-2 pandemic. This is a somewhat surprising but rather encouraging result, with the potential of positively influencing the course of the current pandemic wave.

The antiviral activity of MK-4482 is not affected by mutations in the spike protein and remains active against all variants of SARS-CoV-2. Thus, MK-4482 remains a potent alternative to monoclonal antibody treatment, which is rather vulnerable to mutations in the spike protein (11). Although Omicron already has mutations in the viral polymerase protein (19), the target of MK-4482 antiviral action, the drug remains effective against this VOC, with a significant reduction of viral replication in the upper and lower respiratory tract. It remains to be seen whether future variants that may develop under more widespread clinical use of MK-4482 will acquire further mutations in the polymerase, resulting in reduced drug activity or even loss of drug efficacy. Nevertheless, serial in vitro passage studies of several RNA viruses (20, 21) as well as related coronaviruses (22) under suboptimal levels of the active metabolite of MK-4482 indicate a high genetic barrier of the drug to the acquisition of viral resistance. Selective and targeted clinical use of the drug as well as the consideration of combination therapy with synergistic antivirals or other therapeutic approaches (23) may also help to reduce the development of such drug resistance.

We found a marked discrepancy between viral sgRNA loads and infectious titers for all MK-4482–treated VOCs. Given the mechanism of action of MK-4482 (5) this may not be surprising, as mutated, replication-incompetent viral RNA will still be detected by quantitative RT-PCR analysis but not by infectivity assays. This observation emphasizes the need to consider the specific method of analysis being used to assess antiviral activity, as viral sgRNA loads are often used as the single measure for SARS-CoV-2 replication. Our results strongly suggest that infectious titers are critical for a complete investigation and required to determine the efficacy of antiviral drugs and likely also vaccines. Histopathology serves as an important confirmation for virus replication in target tissues and provides valuable pathologic images.

Our study has limitations. The hamster model does not accurately represent COVID-19 disease for all human age- and comorbidity-associated subpopulations, as the animals only develop mild-to-moderate disease (14, 24, 25), but this is a drawback of most current COVID-19 animal models (26). The more recently identified dwarf hamster model is associated with higher disease severity, but the extremely acute disease progression, with animals reaching clinical endpoints within as quickly as 3 dpi (27), does not parallel the more prolonged course of severe disease in humans (28). In all models, the current intranasal delivery of the challenge virus, while mimicking the most biologically relevant route of infection in humans, may interfere with readout parameters for the upper respiratory tract, negatively affecting analysis of virus replication and shedding. Future transmission studies need to confirm the effect of MK-4482 on virus shedding and, ultimately, transmission to naive animals. A study awaiting peer review has shown an inhibitory effect of MK-4482 on shedding and transmission in the upper respiratory disease ferret model (29), which is encouraging. However, studies investigating the direct transmission from infected to naive animals are more difficult to perform in the hamster (30) and should be targeted for future experiments. The greatest limitation in the current situation is the incomplete experience with MK-4482 in clinical studies against the Omicron VOC. Further studies are underway, and results are expected in the near future. Nevertheless, in vitro studies (11), preclinical data from animal models, such as our study and others (26), and previous clinical trials against earlier SARS-CoV-2 VOCs (6) strongly support the use of MK-4482 for mild-to-moderate COVID cases of Omicron in high-risk adults as well as possibly for postexposure prophylaxis.

In conclusion, our data demonstrate potent efficacy of MK-4482 against the dominant Omicron VOC in a key well-established in vivo COVID-19 disease model. We also demonstrate efficacy of MK-4482 against earlier globally distributed VOCs, identifying MK-4482 as an important broad-acting antiviral intervention for existing and likely future VOCs. The potent efficacy and favorable route of oral administration support further clinical development and broader use of MK-4482 as a key treatment option for SARS-CoV-2 infections in humans.

## Methods

### Biosafety.

SARS-CoV-2 work performed in high biocontainment at the Rocky Mountain Laboratories, NIAID, NIH. Institutional Biosafety Committee–approved Standard Operating Protocols approved by the Rocky Mountain Laboratories Institutional Biosafety Committee were followed for sample removal from biocontainment. Syrian hamsters were group housed in HEPA-filtered cage systems enriched with nesting material and were provided with commercial chow (Teklad Global 16% Protein Rodent Diet) and water ad libitum. Animals were monitored at least twice daily.

### Syrian hamster study design.

Male and female 8- to 10-week-old Syrian Golden hamsters (Envigo) were divided into vehicle (*n* = 15 for Omicron infected, *n* = 11 Alpha, Beta, or Delta infected) or treatment (*n* = 10 for Alpha, Beta, Delta, or Omicron infected; *n* = 9 for the Omicron high-dose infected) groups prior to infection and treatment with MK-4482 (MedChemExpress). MK-4482 was dissolved in DMSO and then resuspended in sterile saline for delivery at 250 mg/kg. Hamsters were treated for 12 hours following infection, and treatment was continued every 12 hours until the completion of the study 84 hours after infection (day 4). Vehicle control animals received the same dosing schedule and volume as VOC infection groups. All groups were infected intranasally with 10^3^ or 10^4^ TCID_50_ (a high dose to account for reduced replication of Omicron, ref. 17, of SARS-CoV-2 via 25 μL/nare). Intranasal inoculations were performed as previously described (14). In brief, hamsters were anesthetized with vaporized isoflurane, and 25 μL inoculum was dropped into each naris by pipette for a total infection volume of 50 μL. Animal weights were collected once daily, and animals were monitored twice daily for disease signs and progression. All procedures were performed on anesthetized animals. Oral swabs were collected on days 2 and 4 after infection. Animals were euthanized on day 4 after infection, and trachea and lung tissues were collected at necropsy for analysis.

### Virus and cells.

The SARS-CoV-2_2hCOV_19_England_204820464_2020 isolate (B.1.1.7; Alpha) was provided by BEI Resources (Manassas). The stock was sequence confirmed and had amino acid changes at ORF1AB (D3725G, 13%) and ORF1AB (L3826F, 18%) when aligned to the GISAID sequence (GISAID no. EPI_ISL_683466). SARS-CoV-2 isolate nCoV-hCoV-19/USA/MD-HP01542/2021 (B.1.351, Beta) was provided by Andrew Pekosz (Johns Hopkins University, Baltimore, Maryland, USA). The virus stock was sequence confirmed and found to have amino acid changes at NSP5 (P252L, 17%) and NSP6 (L257F, 57%) when aligned to the GISAID sequence (GISAID no. EPI_ISL_890360). SARS-CoV-2 variant hCoV-19/USA/KY-CDC-2-4242084/2021 (B.1.617.2, Delta) was obtained through B. Zhou, N. Thornburg, and S. Tong (Centers for Disease Control and Prevention, Atlanta, Georgia, USA). SARS-CoV-2 variant hCoV-19/USA/GA-EHC-2811C/2021 (B.1.1.529, Omicron, EPI_ISL_7171744) was obtained from Mehul Suthar (Emory University, Atlanta, Georgia, USA). Virus stocks for use in the in vivo studies were produced in VeroE6 cells. All viral stocks were sequenced via Illumina-based deep sequencing to confirm identity and exclude any contaminants. Virus propagation was performed in DMEM (MilliporeSigma) supplemented with 2% fetal bovine serum (Gibco), 1 mM L-glutamine (Gibco), and 50 U/mL penicillin and 50 μg/mL streptomycin (Gibco). Vero E6 cells, provided by R. Baric, University of North Carolina, Chapel Hill, North Carolina, USA, were maintained in DMEM (MilliporeSigma) supplemented with 10% fetal calf serum, 1 mM L-glutamine, 50 U/mL penicillin, and 50 μg/mL streptomycin.

### Viral genome detection.

qPCR was performed on RNA extracted from swabs or tissues (30 mg or less) using the QiaAmp Viral RNA kit (Qiagen). A 1-step real-time RT-PCR assay (Qiagen Quantifast) was used to amplify a portion of the E gene to detect subgenomic RNA (15). Dilutions of RNA standards counted by droplet digital PCR were run in parallel and used to calculate viral RNA genome copies. The Rotor-Gene probe kit (Qiagen) was used to run the PCRs according to the instructions of the manufacturer.

### Virus titration assay.

Virus end-point titrations were performed in Vero E6 cells. Briefly, tissue was homogenized in 1 mL DMEM using a TissueLyzer (Qiagen) and clarified by low-speed centrifugation. Cells were inoculated with 10-fold serial dilutions of homogenized lung samples or oral swabs in 100 μL DMEM (MilliporeSigma) supplemented with 2% fetal bovine serum, 1 mM L-glutamine, 50 U/mL penicillin, and 50 μg/mL streptomycin. Cells were incubated for 6 days and then scored for cytopathogenic effects and TCID_50_ was calculated via the Reed-Muench formula.

### Histopathology.

Tissues were embedded in PureAffin paraffin polymer (Cancer Diagnostics) and sectioned at 5 μm for H&E staining. For IHC, tissues were processed using the Discovery Ultra automated stainer (Ventana Medical Systems) with a ChromoMap DAB kit (Roche Tissue Diagnostics, catalog no. 760-159). Specific immunoreactivity was detected using GenScript SARS-CoV-2–specific antiserum (U864YFA140-4/CB2093 NP-1) at a 1:1000 dilution. The secondary antibody was an anti-rabbit IgG polymer (ImmPress-VR, catalog no. MP-6401) from Vector Laboratories.

### Statistics.

Statistical analysis was performed in Prism 9. The Student’s *t* test was 2 tailed, and *P* values of 0.05 were considered significant. The difference in weight, viral load, and infectious titers between study arms was assessed by ordinary 1-way ANOVA (*P* value was set to 0.05).

### Study approval.

The Rocky Mountain Laboratories Animal Care and Use Committee (RML IACUC) approved all animal work under protocol 2020-044-E. Per the RML IACUC, all animal work was performed by certified staff in an Association for Assessment and Accreditation of Laboratory Animal Care International–accredited facility. The institution’s guidelines for animal use, the guidelines and basic principles in the NIH *Guide for the Care and Use of Laboratory Animals* (National Academies Press, 2011), the Animal Welfare Act, the United States Department of Agriculture, and the United States Public Health Service Policy on Humane Care and Use of Laboratory Animals were followed. SARS-CoV-2 work was approved by the Rocky Mountain Laboratories Institutional Biosafety Committee.

## Author contributions

KR, MAJ, and HF contributed to the study design, execution, data analysis, and manuscript writing. AO, MCL, FF, KMW, WFB, and AG contributed experimental support and data analysis. RR and CS contributed to the pathological analysis. HF and MAJ secured funding for the study.

## Version 1 05/17/2022

In-Press Preview

## Version 2 07/08/2022

Electronic publication
